# Prefrontal-amygdala emotion regulation and depression in multiple sclerosis

**DOI:** 10.1093/braincomms/fcac152

**Published:** 2022-06-13

**Authors:** Lil Meyer-Arndt, Joseph Kuchling, Jelena Brasanac, Andrea Hermann, Susanna Asseyer, Judith Bellmann-Strobl, Friedemann Paul, Stefan M Gold, Martin Weygandt

**Affiliations:** Experimental and Clinical Research Center, a Cooperation between the Max Delbrück Center for Molecular Medicine in the Helmholtz Association and Charité Universitätsmedizin Berlin, Berlin, Germany; Charité—Universitätsmedizin Berlin, Corporate Member of Freie Universität Berlin and Humboldt-Universität zu Berlin, Experimental and Clinical Research Center, Lindenberger Weg 80, 13125 Berlin, Germany; Max Delbrück Center for Molecular Medicine in the Helmholtz Association (MDC), Berlin, Germany; Charité – Universitätsmedizin Berlin, corporate member of Freie Universität Berlin, Humboldt-Universität zu Berlin, and Berlin Institute of Health, NeuroCure Clinical Research Center, 10117 Berlin, Germany; Charité – Universitätsmedizin Berlin, corporate member of Freie Universität Berlin, Humboldt-Universität zu Berlin, and Berlin Institute of Health, Department of Neurology, 10117 Berlin, Germany; Charité – Universitätsmedizin Berlin, corporate member of Freie Universität Berlin, Humboldt-Universität zu Berlin, and Berlin Institute of Health, NeuroCure Clinical Research Center, 10117 Berlin, Germany; Charité – Universitätsmedizin Berlin, corporate member of Freie Universität Berlin, Humboldt-Universität zu Berlin, and Berlin Institute of Health, Department of Neurology, 10117 Berlin, Germany; Charité – Universitätsmedizin Berlin, corporate member of Freie Universität Berlin, Humboldt-Universität zu Berlin, and Berlin Institute of Health, NeuroCure Clinical Research Center, 10117 Berlin, Germany; Department of Psychotherapy and Systems Neuroscience, Justus Liebig University Giessen, Germany; Bender Institute of Neuroimaging, Justus Liebig University Giessen, Germany; Center for Mind, Brain and Behavior (CMBB), University of Marburg and Justus Liebig University Giessen, Germany; Charité – Universitätsmedizin Berlin, corporate member of Freie Universität Berlin, Humboldt-Universität zu Berlin, and Berlin Institute of Health, NeuroCure Clinical Research Center, 10117 Berlin, Germany; Experimental and Clinical Research Center, a Cooperation between the Max Delbrück Center for Molecular Medicine in the Helmholtz Association and Charité Universitätsmedizin Berlin, Berlin, Germany; Charité—Universitätsmedizin Berlin, Corporate Member of Freie Universität Berlin and Humboldt-Universität zu Berlin, Experimental and Clinical Research Center, Lindenberger Weg 80, 13125 Berlin, Germany; Max Delbrück Center for Molecular Medicine in the Helmholtz Association (MDC), Berlin, Germany; Charité – Universitätsmedizin Berlin, corporate member of Freie Universität Berlin, Humboldt-Universität zu Berlin, and Berlin Institute of Health, NeuroCure Clinical Research Center, 10117 Berlin, Germany; Experimental and Clinical Research Center, a Cooperation between the Max Delbrück Center for Molecular Medicine in the Helmholtz Association and Charité Universitätsmedizin Berlin, Berlin, Germany; Charité—Universitätsmedizin Berlin, Corporate Member of Freie Universität Berlin and Humboldt-Universität zu Berlin, Experimental and Clinical Research Center, Lindenberger Weg 80, 13125 Berlin, Germany; Max Delbrück Center for Molecular Medicine in the Helmholtz Association (MDC), Berlin, Germany; Charité – Universitätsmedizin Berlin, corporate member of Freie Universität Berlin, Humboldt-Universität zu Berlin, and Berlin Institute of Health, NeuroCure Clinical Research Center, 10117 Berlin, Germany; Charité – Universitätsmedizin Berlin, corporate member of Freie Universität Berlin, Humboldt-Universität zu Berlin, and Berlin Institute of Health, Department of Neurology, 10117 Berlin, Germany; Charité – Universitätsmedizin Berlin, corporate member of Freie Universität Berlin, Humboldt-Universität zu Berlin, and Berlin Institute of Health, Medical Department - Section of Psychosomatic Medicine, Campus Benjamin Franklin, 12203 Berlin, Germany; Charité – Universitätsmedizin Berlin, corporate member of Freie Universität Berlin, Humboldt-Universität zu Berlin, and Berlin Institute of Health, Department of Psychiatry and Psychotherapy, Campus Benjamin Franklin, 12203 Berlin, Germany; Institute of Neuroimmunology and Multiple Sclerosis (INIMS), Center for Molecular Neurobiology Hamburg, Universitätsklinikum Hamburg-Eppendorf, 20251 Hamburg, Germany; Experimental and Clinical Research Center, a Cooperation between the Max Delbrück Center for Molecular Medicine in the Helmholtz Association and Charité Universitätsmedizin Berlin, Berlin, Germany; Charité—Universitätsmedizin Berlin, Corporate Member of Freie Universität Berlin and Humboldt-Universität zu Berlin, Experimental and Clinical Research Center, Lindenberger Weg 80, 13125 Berlin, Germany; Max Delbrück Center for Molecular Medicine in the Helmholtz Association (MDC), Berlin, Germany; Charité – Universitätsmedizin Berlin, corporate member of Freie Universität Berlin, Humboldt-Universität zu Berlin, and Berlin Institute of Health, NeuroCure Clinical Research Center, 10117 Berlin, Germany

**Keywords:** multiple sclerosis, depression, emotion regulation, task-based functional magnetic resonance imaging, tractography

## Abstract

Depression is among the most common comorbidities in multiple sclerosis and has severe psychosocial consequences. Alterations in neural emotion regulation in amygdala and prefrontal cortex have been recognized as key mechanism of depression but never been investigated in multiple sclerosis depression. In this cross-sectional observational study, we employed a functional MRI task investigating neural emotion regulation by contrasting regulated versus unregulated negative stimulus perception in 16 persons with multiple sclerosis and depression (47.9 ± 11.8 years; 14 female) and 26 persons with multiple sclerosis but without depression (47.3 ± 11.7 years; 14 female). We tested the impact of depression and its interaction with lesions in amygdala-prefrontal fibre tracts on brain activity reflecting emotion regulation. A potential impact of sex, age, information processing speed, disease duration, overall lesion load, grey matter fraction, and treatment was taken into account in these analyses. Patients with depression were less able (i) to downregulate negative emotions than those without (*t* = −2.25, *P* = 0.012, *β* = −0.33) on a behavioural level according to self-report data and (ii) to downregulate activity in a left amygdala coordinate (*t* = 3.03, *P*_Family-wise error [FWE]-corrected_ = 0.017, *β* = 0.39). Moreover, (iii) an interdependent effect of depression and lesions in amygdala-prefrontal tracts on activity was found in two left amygdala coordinates (*t* = 3.53, p_FWE_ = 0.007, *β* = 0.48; *t* = 3.21, p_FWE_ = 0.0158, *β* = 0.49) and one right amygdala coordinate (*t* = 3.41, p_FWE_ = 0.009, *β* = 0.51). Compatible with key elements of the cognitive depression theory formulated for idiopathic depression, our study demonstrates that depression in multiple sclerosis is characterized by impaired neurobehavioural emotion regulation. Complementing these findings, it shows that the relation between neural emotion regulation and depression is affected by lesion load, a key pathological feature of multiple sclerosis, located in amygdala-prefrontal tracts.

## Teaser

Consistent with key propositions of the cognitive model for idiopathic depression, Meyer-Arndt *et al.* report that multiple sclerosis depression is characterized by impaired emotion regulation in amygdala and prefrontal cortex. This impairment is modulated by brain lesions, a multiple sclerosis hallmark, located in tracts connecting amygdala and prefrontal cortex.

## Graphical Abstract

**Graphical abstract:**
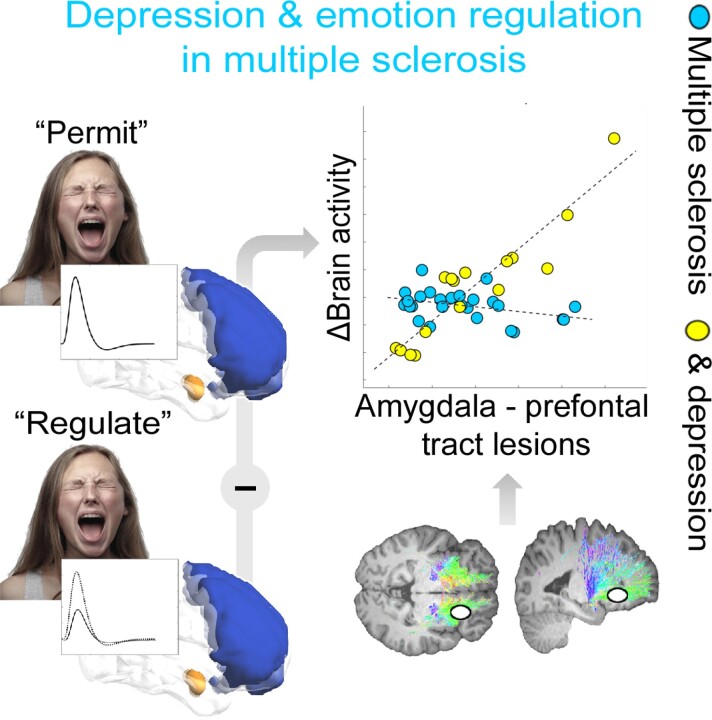

## Introduction

Depression is a frequent comorbidity in multiple sclerosis associated with adverse consequences.^1,2^ The point prevalence of depression in multiple sclerosis has been estimated around 25–30% and is thus ∼2–3-fold higher than in the general population.^3^ Psychosocial consequences of MS-associated depressive disorders comprise lower quality of life, reduced ability to work, earlier retirement, and reduced adherence to multiple sclerosis therapies.^4^ Comorbid depression has been shown to predict multiple sclerosis disease progression, with depressed patients reaching disability milestones significantly faster.^5^ Depressive symptoms can precede multiple sclerosis diagnosis by years, suggesting a potential overlap of pathobiological pathways.^6^ Neural processing of psychological stress, an important contributor to depression,^7^ is related to neurological symptoms^8^ and predicts future regional brain atrophy^9^ and quality of life^10^ in multiple sclerosis. Although several markers of neuroinflammation and neurodegeneration have been linked to multiple sclerosis-associated depression,^6,11–13^ a key factor underlying depression according to its prominent cognitive model,^14^ emotion regulation, was not. According to this model, early life stress and the occurrence of life events in later life are risk factors for the development of depression which is characterized by information processing biases leading to heightened emotional responsivity and impaired emotion regulation. Examples for emotion regulation strategies are reinterpreting (the meaning of emotional stimuli), distancing (detaching from emotional stimuli), and rumination (i.e. repetitive thoughts focusing on one’s negative affect or depressive symptoms, as well as their consequences; see Joormann and Stanton^15^ for an overview). According to the cognitive model, activity alterations in amygdala and prefrontal cortex (PFC), as well as in their interplay are considered as neural substrates of depression-related cognitive processes. Specifically, heightened amygdala activity is related to negative dysfunctional beliefs, cognitive biases,^14^ and the generation of negative affective states.^16^ PFC activity is related to the generation of negative affective states^17^ and the regulation of emotion processing in amygdala.^18,19^ The clinical importance of emotion regulation was indicated by studies showing that persons with depression engage more frequently in less efficient strategies (such as rumination) and appear less able to use efficient ones (such as reinterpreting and distancing).^15,20,21^ The clinical importance of PFC and amygdala as neural substrates of impaired emotion regulation was supported by a variety of neuroimaging studies on depression.^22–24^

Consequently, we investigated the activity in and structural connectivity between amygdala and PFC with an emotion regulation functional MRI task, diffusion-weighted MRI, and tractography in 16 persons with multiple sclerosis and depression (PwMSD) and 26 persons with multiple sclerosis (PwMS) but no depression to study their role for depression in multiple sclerosis. We first analyzed whether task-induced amygdala and PFC activity is related to depression in multiple sclerosis. We hypothesized (→ H1) that PwMSD are less able to downregulate amygdala activity than PwMS.^23^ Given different functional roles of PFC in depression (i.e. generation of depression-related affective states^17^ and top-down inhibitory control of emotion processing in amygdala^18,19^), we hypothesized positive and negative effects of depression on activity in PFC. Given the importance of the interplay between PFC and amygdala for emotion regulation and depression, we then evaluated whether the link between depression and activity in amygdala and PFC coordinates is modulated by lesions in tracts connecting these areas. We hypothesized (→ H2) a positive interaction effect of depression and tract lesions on amygdala activity. Again, due to the functional heterogeneity of PFC, we assumed positive and negative interaction effects for this region’s activity.

## Materials and methods

### Participants

Forty-five persons with relapsing–remitting multiple sclerosis were recruited via the Charité outpatient clinics of the NeuroCure Clinical Research Center and the Experimental and Clinical Research Center. Study participation comprised 2 days (first: clinical assessments, second: MRI) in a 2-weeks period. Recruitment and measurements were performed between May 2017 and December 2018.

Inclusion criteria were age ≥ 18 years, a diagnosis of relapsing–remitting multiple sclerosis according to the McDonald 2010 criteria,^25^ stable treatment with immunomodulatory drugs or no MS-modifying treatment for the last 3 months, and the physical and mental capability to use the test devices without restriction. The following exclusion criteria were applied: a current diagnosis of a psychiatric disorder other than major depression or anxiety disorders, a diagnosis of another neurologic disorder other than multiple sclerosis, a relapse or steroid treatment during the last 4 weeks and MRI-related contraindications.

After application of these criteria, the sample comprised 42 patients with a complete MRI data set. Among the 42 patients, 16 were classified as depressive based on the severity of symptoms, 26 were classified as non-depressed (see clinical assessment). Thus, the group sizes are comparable with those in Erk *et al*.,^23^ a widely renowned study investigating neural emotion regulation in persons with depression and healthy controls with the same task as the present study. All patients provided written informed consent prior to enrolment and received adequate financial reimbursement. The study was conducted in accordance with relevant guidelines (Helsinki Declaration of 1975) and approved by the ethics committee of Charité – Universitätsmedizin Berlin (EA1/208/16). Please note, that functional MRI data of the patients participating in this study assessed at the same day with another task (i.e. on psychological stress) are presented in Brasanac *et al*.^26^

### Clinical assessment

The EDSS^27^ was used to evaluate clinical disability. Assessment of psychiatric disorders for participant exclusion was performed with the German version (5.0.0) of the Mini-International Neuropsychiatric Interview for the fourth edition of the Diagnostic and Statistical Manual of Mental Disorders^28^ (please note that this interview did not exist for the fifth edition at study onset). Severity of depressive symptoms was quantified using the clinician-based MADRS.^29^ Sixteen multiple sclerosis patients met the definition of Herrmann *et al*.^30^ for having at least a mild depression which corresponds to a MADRS-score ≥ 7 and were thus classified as PwMSD. Twelve additionally met the criteria for a major depressive disorder assessed using the Mini-International Neuropsychiatric Interview. The remaining 26 patients not having at least a mild depression (MADRS-score <7) were classified as PwMS. Information processing speed was evaluated with the Symbol Digit Modalities Test (SDMT).^31^ Complementary self-report data were assessed with the German version of the Emotion Regulation Questionnaire (ERQ)^32^ (measuring the habitual use of emotion regulation techniques) and the German version of the State-Trait Anxiety Inventory (STAI)^33^ (measuring anxiety on a trait and state level).

### Emotion regulation task

**Figure 1:**
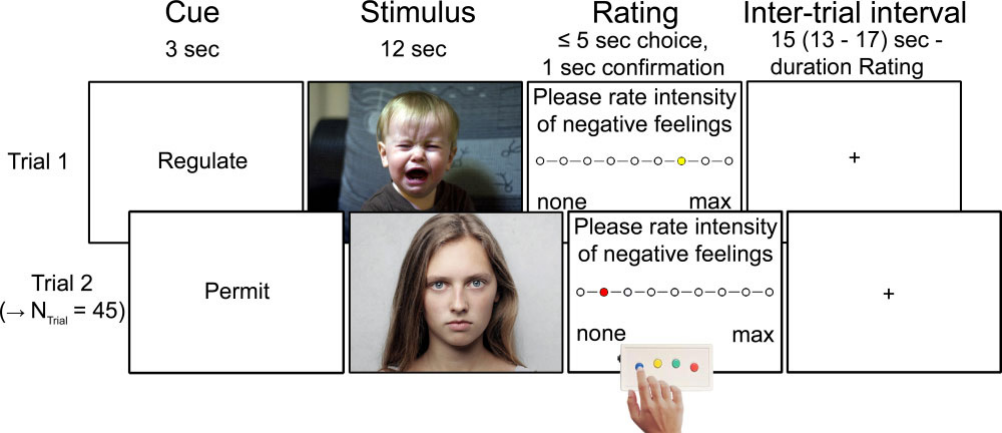
**Functional MRI emotion regulation task.** The figure illustrates 2 of 45 exemplary trials of the task. Please note that the photos depicted here are license-free stock photos used as examples in the figure only; the original pictures of the International Affective Picture System used in the paradigm could not be shown in the figure because they are protected by copyright law.

In this task adapted from the functional MRI study on emotion regulation in depression by Erk *et al*.,^23^ patients were presented with 30 images depicting negative affective content. Fifteen of them were viewed in a non-regulated, permissive fashion, i.e. for which the patients were instructed ‘to look at the presented images and permit feeling potentially induced emotions’ (condition: ‘negative permit’). For the other 15 negative images, patients were instructed to deploy active emotion regulation (i.e. distancing) techniques (‘negative regulate’). Specifically, they were told to imagine ‘that the corresponding stimuli are just pictures’, ‘that you will never experience the depicted events’, ‘that these events are unrelated to you’, or ‘that the pictures are fake’. The task also included the presentation of 15 pictures depicting neutral content, which the patients should view in the non-regulated fashion (‘neutral permit’). This condition enabled investigating emotional responsivity in addition to emotion regulation (see below). All 45 images were taken from the International Affective Picture System^34^ (see Supplementary material for details). Each of the three conditions comprised three images from the five following content-related categories: animals, faces, bodies, objects, and social interaction scenes. A stimulus matching procedure was applied to ensure that psychometric picture properties (i.e. valence and arousal) were comparable for ‘negative regulate’ and ‘negative permit’ and thus to prevent artifactual differences in conditions due to such psychometric differences (see Supplementary material for details). The sequence of the three conditions was determined in a pseudorandom fashion for each patient individually. Each of the 45 pictures was shown in one trial, which started with the presentation of the cue words ‘permit’ or ‘regulate’ (duration: 3 s) to inform the patient whether or not to apply a distancing strategy during perception of the image presented for 12 s after the cue word. Next, the intensity of perceived, image-triggered negative emotions was rated using a 9-point Likert scale (ranging from ‘1 = none’ to ‘9 = maximal’; max. 5 s) with an MRI compatible button box. After rating, the colour of the dot turned from yellow to red for 1 s (or stayed yellow for this time in case of a missing response). Finally, a fixation cross was presented in the inter-trial stage for an average duration of 15 s(range: 13–17) minus the rating duration. Each of the 45 trials had a duration of 30 s, the task one of 22.5 min. Figure 1 provides details.

### MRI sequences

Brain scans were acquired in the Berlin Center for Advanced Neuroimaging with a 3 Tesla whole-body tomograph (Magnetom Trio, Siemens, Erlangen, Germany) using a standard 12-channel head coil. A T_2_*-weighted echo planar imaging functional blood-oxygenation level-dependent imaging sequence comprising 590 images with 38 axial slices covering the whole brain was acquired during the task (voxel resolution = 3 × 3 mm^2^; slice thickness = 3 mm; 0.75 mm gap; TR = 2310 ms; TE = 30 ms; flip angle = 78°; field of view = 192 mm × 192 mm; matrix size = 64 × 64). The total duration of this sequence was 22 min and 43 s. To enable distortion correction of functional images, pairs of spin-echo echo planar imaging reference volumes with opposing phase-encoding directions (anterior-to-posterior, posterior-to-anterior) were acquired prior to the functional scans with matching readout and geometry (i.e. 38 axial slices, voxel resolution = 3 × 3 mm^2^; slice thickness = 3 mm; 0.75 mm gap; TR = 3600 ms; TE = 63 ms; flip angle = 90°; field of view = 192 mm × 192 mm; matrix size = 64 × 64).

To acquire anatomical brain scans, we employed a T_1_-weighted, 3D magnetization-prepared rapid gradient echo sequence of 192 slices covering the whole brain (1 mm isotropic voxels; TR = 1900 ms; TE = 2.52 ms; flip angle = 9°; field of view = 256 × 256 mm^2^; matrix size = 256 × 256) with a duration of 4 min and 26 s. Furthermore, we acquired sagittal T_2_-weighted fluid-attenuated inversion recovery images (176 slices; 1 mm isotropic voxels; TR = 6000 ms; TE = 388 ms; flip angle = 120°; field of view = 256 × 256 mm^2^; matrix size = 256 × 256; 7 min 44 s duration).

Finally, we measured a diffusion-weighted imaging (DWI) sequence with 60 axial slices covering the whole brain (2.3 mm isotropic voxels; TR = 7500 ms; TE = 85 ms; flip angle = 90°; field of view = 220 × 220 mm^2^; matrix size 96 × 96, bandwidth = 1336 Hz/pixel, diffusion weighting at a high b = 1000 s/mm^2^ along 64 directions) with a duration of 8 min and 32 s. Again, we acquired pairs of spin-echo echo planar image reference volumes with opposing phase-encoding directions (anterior-to-posterior, posterior-to-anterior) in advance of the DWI scans with matching readout and geometry to enable distortion correction of DWI images (i.e. 60 axial slices covering the whole brain; 2.3 mm isotropic voxels; TR = 7500 ms; TE = 85 ms; flip angle = 90°; field of view = 220 × 220 mm^2^; matrix size 96 × 96).

### MRI preprocessing

#### Functional scans

The standard functional preprocessing performed was conducted with SPM12 (Wellcome Trust Centre for Neuroimaging, Institute of Neurology, UCL, London UK ­ http://www.fil.ion.ucl.ac.uk/spm) and associated toolboxes. It comprised a head motion correction of patients’ 590 functional scans, coregistration of two spin-echo echo planar imaging volumes to average functional images for distortion correction, distortion correction, slice acquisition correction, and linear coregistration to T_1_-weighted images. Finally, the functional images were mapped to the anatomical standard space defined by the MNI, ^35^ spatially smoothed (8 mm full-width at half maximum Gaussian kernel), and a temporal high-pass filter (128 s cut-off) was applied. See also Supplementary material.

#### Diffusion-weighted scans/tractography

Mapping of tracts connecting both amygdalae and PFC was performed with MRtrix3,^36^ necessary preprocessing steps with SPM12. The latter comprised head motion correction and eddy current removal, distortion correction, computation of patient-specific masks for CSF and masks for left amygdala, right amygdala, and PFC. Tractography of fibres connecting left amygdala and PFC and right amygdala and PFC were determined separately. Finally, we computed maps assessing the number of streamlines per voxel. These maps together with individual lesion masks were used to determine patient-specific summary parameters of amygdala-prefrontal tract lesion load which we refer to as ‘Strategic LL’ in the following. Specifically, this marker was computed as proportion of the sum of streamlines included in lesion voxels in amygdala-prefrontal tracts relative to the sum of streamlines in all amygdala-prefrontal tract voxels. This was done separately for the tract connecting left amygdala with PFC and for the tract connecting right amygdala and PFC. The sum of the proportions for left and right amygdala-prefrontal tracts finally served as Strategic LL marker. See also preprocessing of anatomical and DWI scans in the Supplementary material for further details.

### Statistical analysis

#### Structural brain integrity

We determined group differences in structural brain integrity as reflected by the overall GM fraction, overall T_2_-weighted lesion load, and Strategic LL. Prior to the analyses, we performed an arcsine transformation of the GM fraction and Strategic LL data and a log-transform of the overall lesion load data to a to account for the frequent non-normal distribution of proportional data^37^ (i.e. GM fraction & Strategic LL) or whole-brain lesion volume data.^38^ Sex, age, disease duration, and a constant served as covariates of no interest (CNI) in all analyses. We assumed that PwMS have better structural brain health than PwMSD and conducted directed tests accordingly (α = 0.05).

#### Psychological emotion regulation

To evaluate group differences in psychological emotion regulation, we conducted linear mixed model (included in Matlab 2014a; MathWorks, Natick, Massachusetts, USA) regression analyses (e.g. Weygandt *et al*. ^39^) based on rating data acquired during the functional MRI task. Because the recording of these data failed in four patients, this was done based on the data of 38 patients. Given that the success in emotion regulation can only be determined by relating feelings reported during regulation to those during an unregulated reference condition, group differences were evaluated by determining the interaction effect condition (‘negative regulate’ versus. ‘negative permit’) × group by implementing a corresponding regressor as covariate of interest in the linear mixed model. CNI were condition, group, overall T_2_-weighted lesion load, Strategic LL, group × Strategic LL, disease duration, sex, age, and constant. A constant was added as random CNI. Permutation testing (5000 permutations) was used for inference. In a complementary analysis, we tested group differences in basic emotional responsivity to negative stimuli (compare Riccelli *et al*.^40^) by determining the interaction effect condition (‘negative permit’ versus. ‘neutral permit’) × group. A significance threshold for directed tests (α = 0.05) was applied in each comparison as we assumed that PwMSD have worse regulation abilities (and higher emotional responsivity) than PwMS. For a corresponding analysis controlling for conceivable treatment effects by constraining the analysis to those patients who did receive multiple sclerosis treatment but no antidepressants and by modelling interfon multiple sclerosis therapy as additional CNI, see Supplementary material.

#### Regional brain activity

A proof-of-principle analysis testing the suitability of our functional MRI task for measuring neural emotion regulation was conducted first. Subsequently, we tested H1 and H2. For these purposes, voxel-wise intra-participant brain activity was modelled with a linear regression model using SPM12 beforehand. The model was based on nine boxcar regressors, three for each condition and trial event (i.e. word cue presentation, 3 s; stimulus presentation, 12 s; rating, max. 6 s). Before inclusion in the model, the boxcar regressors were convolved with a haemodynamic response function. Head motion parameters were included as CNI. Given that evaluating emotion regulation necessitates comparing processes during active regulation to those during an unregulated reference condition, voxel measures for emotion regulation were computed as difference between regression coefficients for ‘negative regulate’ minus those for ‘negative permit’. These voxel difference maps were then entered into group-level analyses.

In particular, to evaluate the ability of the task to measure neural emotion regulation, we tested the main effect of this condition on brain activity by applying one-sample *t*-tests based on the voxel difference maps. Whole-brain lesion load, disease duration, age, and sex were included in these analyses as CNI. An family-wise error (FWE)-corrected significance threshold of α_FWE_ = 0.05 was applied in this analysis. The regression model employed for testing H1 and H2 comprised two covariates of interest: A main effect regressor for depression coding ones for PwMSD and zeros for PwMS (→ H1) and a regressor reflecting the interaction between depression and Strategic LL computed as the element-wise product of the main effect regressors for depression and Strategic LL (→ H2). The main effect regressors for Strategic LL, sex, age, disease duration, overall T2-weighted lesion load plus constant served as CNI. Importantly, overall T2-lesion load was included to ensure that interaction effects potentially found for MADRS × Strategic LL do not simply reflect lesions distributed across the whole brain.

SnPM13, an SPM toolbox using permutation testing (5000 permutations) and the maximum statistic procedure to adjust for multiple tests or FWE (threshold: α_FWE_ = 0.05) respectively (www2.warwick.ac.uk/fac/sci/statistics/staff/academicresearch/

Nichols/snpm), was used for inference. Given the strong indication for an amygdala and PFC involvement in emotion processing and depression,^14,15,22,23,41^ the proof-of-principle analysis and the analyses for testing H1 and H2 were conducted once for all 41 bihemispheric amygdala voxels and once for all 4034 PFC voxels. (The Supplementary material provides a visualization of evaluated coordinates.) We report the *t*-statistic and FWE-corrected type I error rate for the peak voxel, the cluster size, and standardized regression coefficient *β* as effect size measure.^42^ According to Acock^43^, |*β*| <0.2 corresponds to a weak, 0.2 ≤ |*β*| <0.5 to a moderate, and |*β*| ≥ 0.5 to a strong effect. Please note that the Supplementary material includes two analyses complementing the functional MRI analyses described above. First, all analyses described for emotion regulation were repeated for emotional responsivity. These analyses relied on voxel contrast maps coding for the differences in regression coefficients for ‘negative permit’ minus ‘neutral permit’. Second, the Supplementary material also includes an analysis testing associations between both neural emotion processing parameters and depression and amygdala-PFC lesions that additionally controlled for putative treatment effects.

### Data availability

Anatomical MRI images will not be made available due to privacy issues of clinical data. Moreover, all data used in this research were collected subject to the informed consent of the patients. Consequently, access to data other than anatomical MRI will be granted by the corresponding author on request only in line with that consent, subject to approval by the project ethics board and under a formal Data Sharing Agreement.

## Results

### Clinical and demographic sample characteristics

Thirty-one patients received a multiple sclerosis modifying treatment (6 Dimethylfumarat, 6 Fingolimod, 5 Glatirameracetat, 9 Interferon, 4 Teriflunomid, 1 Alemtuzumab), three antidepressants (2 tetracyclic antidepressants, 1 selective serotonin reuptake inhibitors). See also Table 1.

**Table 1:** Demographic and clinical participant characteristics

*Group*	*Sex* *(f./m.)*	*Age* *(years)*	*ERQ* *Reappraisal* *(pts.)*	*ERQ* *Suppression* *(pts.)*	*MADRS* *(pts.)*	*SDMT* *(#corr. trials)*	*STAI-State* *(pts.)*	*STAI-* *Trait* *(pts.)*	*EDSS* *(pts.)*	*Disease duration* *(years)*
	#	MN	MN	MN	MN	MN	MN	MN	MD	MN
		SD	SD	SD	SD	SD	SD	SD	RG	SD
*Pw* *MSD*	14	47.9	28.7	14.9	17.6	54.1	46.2	48.6	2.5	15.2
	2	11.8	4.74	3.46	6.46	8.26	9.14	8.16	1.0-6.0	10.3
*PwMS*	14	47.3	27.7	13.4	1.31	53.4	31.5	32.5	2.5	12.8
	12	11.7	5.95	5.57	1.19	10.5	6.28	5.56	0.0 –6.0	7.8
	χ^2^	*t*	*t*	*t*	*t* ^§^	*t*	*t*	*t*	*T*	*t*
	*P*	*P*	*P*	*P*	*P*	*P*	*P*	*P*	*P*	*P*
	4.73	0.15	0.54	0.96	12.6	0.23	6.19	7.64	1.32	0.84
	0.03	0.88	0.59	0.34	8·10^−16^	0.59	10^−7^	10^−9^	0.10	0.20

Undirected tests were conducted for sex, age, and both ERQ measures (i.e. *P*-values corresponded to two-sided tests), directed tests for all other measures (i.e. *P*-values correspond to one-sided tests). **ERQ – Reappraisal**, habitual application of the emotion regulation strategy ‘reappraisal’. **ERQ – Suppression**, habitual application of the emotion regulation strategy ‘suppression’; **MADRS,** Montgomery-Asberg Depression Rating Scale; **SDMT,** Symbol Digit Modalities Test. **STAI, State** – State anxiety. **STAI – Trait** – Trait anxiety; **corr,** correct; **f./m**., female/male; **pts.,** points. **^§^**Please note that we report measures of inferential statistics for MADRS group differences only for the sake of completeness. From a statistical viewpoint, the parameters reported are meaningless as the group assignment was performed based on MADRS.

### Statistical analysis

#### Structural brain integrity

**Figure 2:**
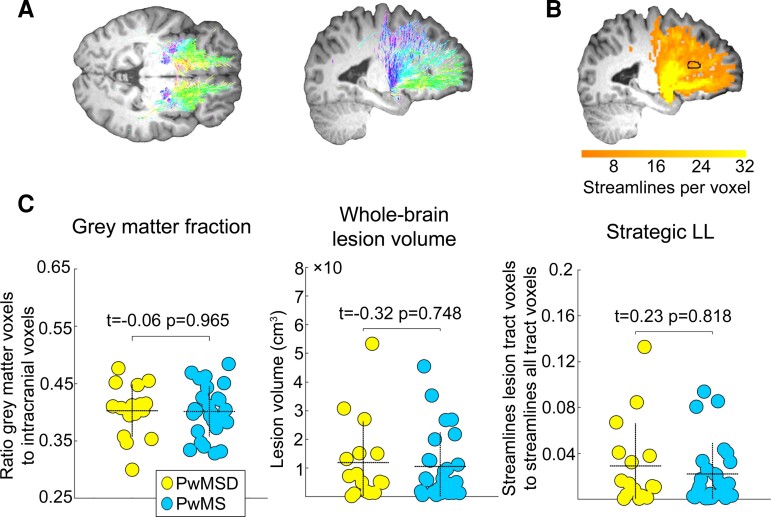
**Structural brain integrity parameters per group.** **A** and **B** illustrate the computation of the Strategic LL parameter, which reflects the affectedness of tracts connecting both amygdalae and PFC by focal brain lesions. Specifically, **A** shows streamlines in tracts connecting left and right amygdala with PFC for an exemplary participant. The different colours denote the streamline directionality. **B** shows a mapping of these streamlines to voxel space. The orange colour range reflects the number of streamlines per voxel. The dashed horizontal lines in each graph of **C** depict the mean, the dashed vertical lines the standard deviation of each parameter for each group. Please note that the seeming small mismatch between the reported *t*-statistics and the relations among group means depicted in the scatter graphs by the dashed horizontal lines across parameters follows from the fact that the *t*-statistics were computed based on transformed parameter values using regression models including CNI (see Methods), whereas the scatter graphs depict raw parameter values.

PwMSD did not differ from PwMS in terms of structural brain integrity – in none of the three measures (GM fraction: *t* = −0.06, *P* = 0.956, *β* = −0.01; overall T_2_-weighted lesion load: *t* = −0.32, *P* = 0.748, *β* = −0.05; Strategic LL: *t* = 0.23, *P* = 0.818, *β* = 0.04). See Fig. 2 for an illustration.

#### Psychological emotion regulation

**Figure 3:**
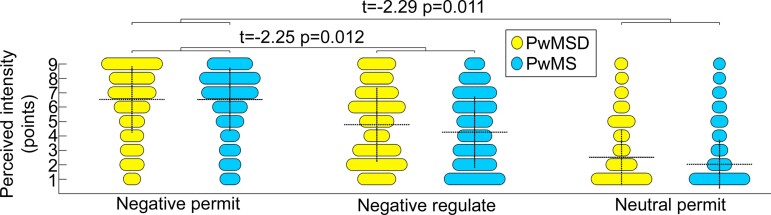
**Psychological emotion regulation during perception of negative stimuli**. The dashed horizontal lines depict the mean, and the dashed vertical lines depict the standard deviation of stimulus ratings obtained during the task for each combination of condition and group. The statistical parameters presented at each bracket correspond (from left to right) to the *t*-statistic, *P*-value, and standardized regression coefficient *β* for significant interaction effects. In addition to results for emotion regulation, the figure also shows the results for emotional responsivity obtained in the complementary analysis.

According to the rating data, PwMSD were significantly less able to downregulate their negative emotions than PwMS (*t* = −2.25, *P* = 0.012, *β* = −0.33). Complementary analyses showed that PwMSD were characterized by a higher emotional responsivity than PwMS (*t* = −2.29, *P* = 0.011, *β* = −0.22) or relatively less reduction of perceived negative feelings during neutral permit respectively (which explains the negative *t*-statistic). Figure 3 provides further details.

#### Regional brain activity

**Figure 4:**
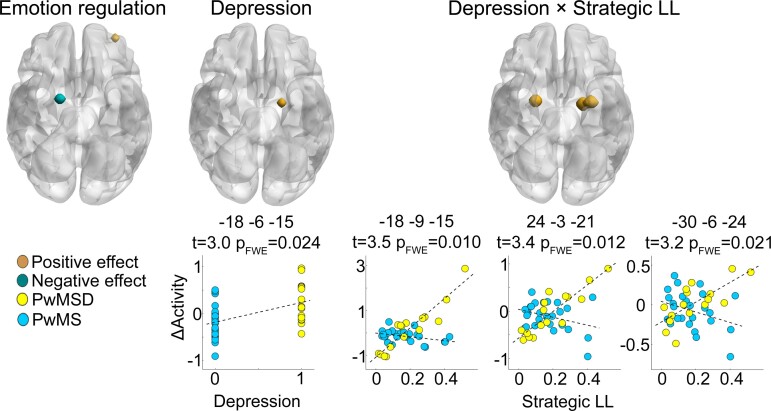
**Regional brain activity.** The render brain in the left shows an amygdala area significantly deactivated during negative regulate versus negative permit across all patients (i.e. a main effect of emotion regulation) and a PFC area showing the opposite behaviour. For the amygdala area depicted in the middle render brain, PwMSD are significantly less able to downregulate activity during negative regulate as compared with negative permit than PwMS (main effect of depression). For the peak coordinate in this area, this is also illustrated in the (left) scatter graph below. The right render brain highlights amygdala areas whose differential activity (‘ΔActivity’; i.e. for negative regulate minus negative permit) depends on the interaction between depression and lesions located in amygdala-prefrontal lesions (interaction effect: Depression × Strategic LL). The three scatter graphs below the right render brain illustrate this for the three peak coordinates in these larger areas. The parameters above each scatter graph denote the coordinates of a given peak coordinate in MNI space, the *t*-statistic and the Type I error rate corrected for family-wise error.

The proof-of-principle analysis testing main effects of emotion regulation revealed a significant negative impact of this factor on activity in a right amygdala coordinate (MNI: 18, −3, −15; *t* = 3.00, p_FWE_ = 0.034) and a significant positive effect on activity in a left middle frontal gyrus coordinate (MNI: −33, 54, −3; *t* = 4.04, p_FWE_ = 0.049). Moreover, supporting H1, PwMSD were less able to downregulate activity in a left amygdala coordinate (MNI: −18, −6, −15; *t* = 3.03, p_FWE_ = 0.017, *β* = 0.39) or relatively less able to reduce amygdala activation during negative regulate relative to negative permit respectively (which explains the positive *t*-statistic). Supporting H2, an interdependent effect of depression and amygdala-prefrontal tract lesions on activity was found in two left amygdala coordinates (MNI: −18, −9, −15; *t* = 3.53, p_FWE_ = 0.007, *β* = 0.48; MNI: −30, −6, −24; *t* = 3.21, p_FWE_ = 0.0158, *β* = 0.49) and one right amygdala coordinate (MNI: 24, −3, −21; *t* = 3.41, p_FWE_ = 0.009, *β* = 0.51). See Table 2 and Fig. 4 for further details.

**Table 2:** Brain activity affected by emotion regulation, depression, and amygdala-PFC tract lesions

*Effect/region*	*x*	*y*	*z*	*Cluster* *Size*	*t*	*p_FWE_*	*Β*
*Emotion regulation*							
*Amygdala*	18	−3	−15	54	−3.00	0.033	*
*Middle frontal gyrus*	−33	54	−3	27	4.03	0.049	*
*Depression*							
*Amygdala*	−18	−6	−15	54	3.03	0.024	0.39
*Depression* × *Strategic LL*							
*Amygdala*	−18	−9	−15	189	3.53	0.010	0.48
*Amygdala*	24	−3	−21	108	3.41	0.012	0.51
*Amygdala*	−30	−6	−24	189	3.21	0.021	0.49

x, y, and z correspond to coordinates of voxels in the MNI space with peak effect size in a cluster of significant voxels. The cluster size corresponds to the volume of significant voxels according to α_FWE_ = 0.05 in mm^3^. *Please note that the regression coefficient for the intercept (i.e. the constant regressor of interest in the analysis of main effects of emotion regulation) cannot be standardized due to the lack of variation in this variable. Consequently, we cannot report the standardized regression coefficient as effect size measure for this proof-of-principle analysis.

## Discussion

Depression is one of the most common comorbidities in multiple sclerosis and has severe psychosocial consequences. Clinical and functional brain imaging studies have identified alterations in emotion regulation as a key mechanism of depression.^15,22,24^ However, emotion regulation has never been investigated in multiple sclerosis depression. Thus, we investigated neurocognitive emotion regulation in persons with multiple sclerosis with and without comorbid depression using a functional MRI task widely established for this purpose.

Analyzing psychological emotion regulation based on self-report data acquired during the task revealed that PwMSD have a reduced ability to downregulate negative emotions compared to PwMS. Given the application of the distancing strategy in our study and findings made in persons suffering from depression alone,^20^ depressed multiple sclerosis patients thus seem to have difficulties in detaching themselves from stimulus-induced negative emotional thoughts. Moreover, compatible with Riccelli *et al*.,^40^ a complementary analysis showed that PwMSD were also characterized by a higher emotional responsivity on a psychological level than PwMS. The fact that depressed multiple sclerosis patients thus show signs of both key components of Beck’s depression model,^14^ reduced emotion regulation and heightened emotional responsivity, underscores the importance of cognitive factors.

A proof-of-principle analysis testing main effects of emotion regulation on regional brain activity revealed an important role of amygdala and PFC for this condition across all patients. Specifically, the overall property of the ‘negative regulate’ condition to attenuate negative affect on a neural level was indicated by the fact that amygdala, a region shown to generate negative emotions,^16^ activated less pronounced during that condition than during the reference condition ‘negative permit’. Furthermore, consistent with the prominent role of dorsolateral prefrontal cortex for emotion regulation,^19^ activity in a coordinate of this area was significantly elevated during ‘negative regulate’ as compared to ‘negative permit’ across all patients. In addition, activation reflecting emotional responsivity spanned wide portions of PFC and amygdala as determined in a supplementary analysis. Together, these results underline the basic suitability of our task to trigger and measure neural emotion processing.

Testing H1 showed that depression in multiple sclerosis is accompanied by a lower ability to downregulate left amygdala activity. This is in line with non-multiple sclerosis work linking amygdala activity to negative dysfunctional beliefs and cognitive biases^14^ and understandable when considering important functional roles of amygdala, i.e. to generate affective states, analyse potential threats, and amplify affective memories.^16^ Consequently, consistent with our first hypotheses (H1), this finding shows that impaired emotion regulation is related to depression in multiple sclerosis in a similar fashion as in idiopathic depression.

Furthermore, consistent with H2 and previous work on amygdala^16^ and PFC functions (generation of depression-related affective states^17^; inhibitory amygdala control^18,19^), the analysis of brain activity showed that depression in multiple sclerosis and amygdala-PFC tract lesions have an interdependent impact on neural emotion regulation in both amygdalae. Specifically, whereas neural emotion regulation (i.e. differential activity for negative regulate minus negative permit) was nearly unaffected by lesions in amygdala-prefrontal tracts in PwMS, a significantly more pronounced positive association was observed in PwMSD. Importantly, this finding suggests that (i) amygdala emotion processing does not require external (i.e. PFC) fine-tuning in non-depressed multiple sclerosis patients, (ii) but it does in depressed ones and (iii) that the ability to do so depends on the structural integrity of amygdala-prefrontal tracts. Consequently, this finding suggests a way by which brain lesions, key neuropathologic factors of multiple sclerosis, could contribute to a depression-related impairment in neural emotion regulation in multiple sclerosis.

An aspect of the study that should be discussed is that of potential confounding factors. Several aspects argue against the possibility that such factors seriously affected our results. First, a supplementary analyses repeating the functional MRI analysis of the main text that did additional control for treatment effects also identified the region in left amygdala (peak coordinate MNI: −18, −6, −15) showing a main effect of depression and an interaction effect depression × Strategic LL in the main-text analyses which not controlled for treatment. Second, a variety of clinico-demographic, neurological, and neuroradiographic multiple sclerosis severity markers were included in the respective regression analyses in the main text as CNI. Third, the application of permutation testing in all major analyses ensures that the results do not reflect methodological artifacts in that sense that they can not rely on a misfit between sample and parametric distributions because permutation testing does not rely on such distributions.^44^

Another point worth mentioning is that non-invasive DWI-based fibre tractography was used to determine the Strategic LL marker and that this method has been criticized in several recent studies (e.g. Maier-Hein *et al*.^45^). In particular, although these authors report a very good sensitivity (i.e. 90% of fibres validated by a radiologist and included in a synthetic connectome as ground truth were identified in a multi-group challenge), they also found that tractography reveals a lot of false positive/inexistent fibres—a fact that was not adequately recognized beforehand due to the lack of a ground truth. Thus, caution might be recommended when interpreting parameters derived from DWI-tractography. At the same time, however, Maier-Hein *et al*.^45^ show that the accuracy of tractography varies across anatomical tracts and results of Folloni *et al*.^46^ suggest that accuracy for amygdala-prefrontal tracts is good. Specifically, Folloni *et al*.^46^ applied DWI-based tractography in macaques and humans to map amygdala-prefrontal tracts. Importantly, measuring DWI-tractography in the macaque allowed us to compare the results obtained to ground truth data obtained by invasive tract tracers macaque studies (e.g. Fudge *et al*.^47^). Folloni *et al*.^46^ found that the tracts identified in the macaque monkey by DWI-tractography closely overlapped with those found by invasive tracers. Simultaneously, also the overlap between amygdala-prefrontal tracts in macaques and humans identified by DWI-tractography was very strong. Thus, given that also the retest reliability of streamline counts identified by DWI-based global tractography is high,^48^ the findings of Folloni *et al*.^46^ suggest that the Strategic LL marker based on voxel-streamline counts provides a clinically meaningful parameter of amygdala-prefrontal tract damage.

A further aspect worth discussing relates to the fact that contrary to emotion regulation assessed within the functional MRI task, habitual ERQ did not differ significantly between groups; neither for the reappraisal nor for the suppression scale of the ERQ. This difference between measurement instruments may derive from the immediate and more specific way of task-based emotion regulation assessment. In contrast to questionnaires, our task-based strategy does not rely on long-term memory, which could be biased, but asks for immediate responses to experimentally controlled affective pictures (including drastic images showing mutilated bodies, and so on). Thus, subjective inferences on emotions induced by affective picture perception may rely stronger on ‘measuring’ a specific response to specific external stimuli as compared to when such inferences are made in response to a questionnaire item. Another explanation may be that the instruments conceptualize emotion regulation differently, whereas patients were explicitly instructed to employ the distancing reappraisal strategy in the functional MRI task, reappraisal as assessed by ERQ is defined less specifically. Importantly, these two differences are well captured by the state-trait concept defined to describe the personality of an individual (e.g. Schmitt & Blum^49^). In this concept, traits denote context-independent cognitive, affective, and behavioural patterns of a person that are stable across different situations, whereas states reflect patterns occurring in (immediate) response to specific situations that vary across situations. The individual relevance of state and trait markers is indicated by the fact that various self-report instruments provide individual scales for state and trait aspects of a concept (e.g. the STAI) and by neuroimaging studies identifying different neural foundations of state and trait markers (e.g. Saviola *et al*.^50^). Thus, the lack in concordance between task-based and ERQ-based assessment of emotion regulation does not question our results especially given the application of an established experimental fMRI task and the fact that the results were obtained by testing hypotheses inferred from a substantial body of prior research.^14,15,41^

Moreover, the lack of an idiopathic depression group prevented evaluating whether the effects observed with regard to depression were specific to multiple sclerosis, which is a drawback of the study. However, the first major goal of this work was to clarify whether a key process of idiopathic depression (i.e. neural emotion regulation in key affective brain regions) also applies to multiple sclerosis depression. This question was addressed as we were surprised by the small number of multiple sclerosis studies investigating crucial idiopathic depression mechanisms despite the fact that depression is considered highly important for multiple sclerosis.^1,2^ Consequently, a missing idiopathic depression group did not prevent addressing our key research goal.

Another limitation of the study is that group sizes were not determined based on an priori power analysis. Given that the numbers of participants per group in our study are compatible to or larger than those in Erk *et al*.,^23^ and that we used the same task as these authors to study emotion regulation, and that our results are consistent with findings of a large number of non-multiple sclerosis depression studies, this aspect appears not to question our results in a serious manner.

To conclude, the study shows an important role of neurocognitive emotion regulation for multiple sclerosis depression and how brain damage characteristic for multiple sclerosis interacts with emotion regulation in depression and multiple sclerosis. Moreover, our findings advocate the application of cognitive therapies aiming to improve emotion regulation skills (e.g. Renna *et al*.^51^) to improve affective symptoms in multiple sclerosis.

## Supplementary Material

fcac152_Supplementary_Data

## Abbreviations

CNI =: covariates of no interest
DWI =: diffusion-weighted imaging
EDSS =: Expanded Disability Status Scales
ERQ =: Emotion Regulation Questionnaire
FWE =: family-wise error
GM =: grey matter
H1, H2 =: first and second hypothesis
MADRS =: Montgomery-Asberg Depression Rating Scale
MNI =: Montreal Neurological Institute
PFC =: prefrontal cortex
PwMS =: persons with multiple sclerosis
PwMSD =: persons with multiple sclerosis and depression
SDMT =: Symbol Digit Modalities Test
STAI =: State-Trait Anxiety Inventory
Strategic LL =: Strategic Lesion Load
