# Supplementary material for: Prefrontal-amygdala emotion regulation and depression in multiple sclerosis
Source: Brain Commun. 2022 Jun 13;4(3):fcac152. doi: 10.1093/braincomms/fcac152 (PMC9218780; doi:10.1093/braincomms/fcac152)
Supplement: fcac152_Supplementary_Data [file fcac152_supplementary_data.docx]

**Prefrontal-amygdala emotion regulation
and depression in multiple sclerosis**

Lil Meyer-Arndt^a, b, c, d, e^, Joseph Kuchling^d, e^, Jelena Brasanac^d^,

Andrea Hermann^f, g, h^, Susanna Asseyer^d^, Judith Bellmann-Strobl^a, b, c, d^,

Friedemann Paul^a, b, c, d, e, *^, Stefan M. Gold^i, j, k, *^, and Martin Weygandt^a, b, c, d, *,§^

**Supplementary material**

^a^ Experimental and Clinical Research Center, a Cooperation between the Max Delbrück Center for Molecular Medicine in the Helmholtz Association and Charité Universitätsmedizin Berlin, Berlin, Germany

^b^ Charité—Universitätsmedizin Berlin, Corporate Member of Freie Universität Berlin and Humboldt-Universität zu Berlin,

Experimental and Clinical Research Center, Lindenberger Weg 80, 13125 Berlin, Germany

^c^ Max Delbrück Center for Molecular Medicine in the Helmholtz Association (MDC), Berlin, Germany

^d^ Charité – Universitätsmedizin Berlin, corporate member of Freie Universität Berlin, Humboldt-Universität zu Berlin, and Berlin Institute of Health, NeuroCure Clinical Research Center, 10117 Berlin, Germany.

^e^ Charité – Universitätsmedizin Berlin, corporate member of Freie Universität Berlin, Humboldt-Universität zu Berlin, and Berlin Institute of Health, Department of Neurology, 10117 Berlin, Germany.

^f^ Department of Psychotherapy and Systems Neuroscience, Justus Liebig University Giessen, Germany

^g^ Bender Institute of Neuroimaging, Justus Liebig University Giessen, Germany

^h^ Center for Mind, Brain and Behavior (CMBB), University of Marburg and Justus Liebig University Giessen, Germany

^i^ Charité – Universitätsmedizin Berlin, corporate member of Freie Universität Berlin, Humboldt-Universität zu Berlin, and Berlin Institute of Health, Medical Department - Section of Psychosomatic Medicine, Campus Benjamin Franklin, 12203 Berlin, Germany.

^j^ Charité – Universitätsmedizin Berlin, corporate member of Freie Universität Berlin, Humboldt-Universität zu Berlin, and Berlin Institute of Health, Department of Psychiatry and Psychotherapy, Campus Benjamin Franklin, 12203 Berlin, Germany.

^k^ Institute of Neuroimmunology and Multiple Sclerosis (INIMS), Center for Molecular Neurobiology Hamburg, Universitätsklinikum Hamburg-Eppendorf, 20251 Hamburg, Germany

Short title: Emotion regulation in multiple sclerosis.

Key words: Multiple Sclerosis; Depression; Emotion Regulation; Task-based functional Magnetic Resonance Imaging; Tractography.

*These authors contributed equally

^§^Corresponding author. Charité – Universitätsmedizin Berlin, Charitéplatz 1, 10117 Berlin, Germany. Email: [martin.weygandt@charite.de](mailto:martin.weygandt@charite.de)

**Materials and methods**

**fMRI emotion regulation task**

International Affective Picture System (IAPS) picture numbers

The 45 IAPS pictures with the following numbers were used for the fMRI task: 1390, 1560, 1675, 2102, 2104, 2210, 2301, 2375.1, 2390, 2393, 2397, 2441, 2570, 2800, 2830, 2900, 3015, 3063, 3102, 3120, 3130, 3131, 5395, 6313, 6315, 6520, 6560, 7595, 7620, 9140, 9181, 9183, 9185, 9187, 9332, 9421, 9425, 9428, 9570, 9600, 9610, 9611, 9620, 9911, 9920.

Stimulus matching procedure

To ensure that psychometric picture properties were comparable across conditions (and thus could not induce artifactual effects of condition on emotion processing), a stimulus matching procedure was applied. For this purpose, we divided the 30 negative images in two sets of 15 images with comparable valence and arousal for each content category separately using an iterative pairwise stimulus matching procedure. Specifically, this iterative procedure selected three pairs of images from the six pictures included in each of the five categories (animals, faces, bodies, objects, and social interaction scenes) based on the minimum Euclidean distance between images in a two-dimensional space spanned by the pictures’ valence and arousal. Thus, the algorithm first searched for the two images with the minimum distance regarding valence and arousal and assigned these images to the first pair. Then, it selected two of the remaining four images repeating the procedure; the remaining two images formed the third pair. Using this procedure, the average valence and arousal between the two negative image sets was highly compatible. This is shown in Supplementary Table 1 below. This table also documents physical stimulus properties (i.e., brightness, contrast, and average image frequency), which were comparable across image sets.

**Supplementary Table 1. Affective and physical stimulus properties by picture set.**

|  | Negative – set 1 | Negative – set 2 | Neutral | Negative – set 1 vs.  negative – set 2 | Negative – set 1 vs.  neutral | Negative – set 2 vs.  neutral |
| --- | --- | --- | --- | --- | --- | --- |
|  | MN (SD) | MN (SD) | MN (SD) | t (p) | t (p) | t (p) |
| Valence  (points) | 2.15  (0.45) | 2.07  (0.43) | 4.98  (0.49) | 0.47  (0.640) | -16.56  5.4×10^-16^ | -17.28  1.8×10^-16^ |
|  |  |  |  |  |  |  |
| Arousal  (points) | 5.91  (0.72) | 5.98  (0.60) | 3.81  (0.89) | -0.27  (0.789) | 7.13  9.2×10^-8^ | 7.85  1.5×10^-8^ |
|  |  |  |  |  |  |  |
| Brightness  (a.u.) | 0.389  (0.078) | 0.417  (0.087) | 0.393  (0.137) | -0.94  0.357 | -0.01  0.923 | 0.58  0.569 |
|  |  |  |  |  |  |  |
| Contrast  (a.u.) | 0.260  (0.040) | 0.260  (0.048) | 0.273  (0.053) | -0.00  0.997 | -0.77  0.446 | -0.71  0.481 |
|  |  |  |  |  |  |  |
| Average image frequency  (a.u.) | 0.0038  (5.2 ×10^-4^) | 0.0037  (4.7 ×10^-4^) | 0.0038  (5.0 ×10^-4^) | 0.35  0.726 | 0.07  0.943 | -0.28  0.778 |

In a second step, the images of the two negative sets were assigned to “negative permit” and “negative regulate” trials in a pseudorandom fashion to the patients across three groups (PwMSD, PwMS, and HC). This yielded a comparable distribution of the two negative picture sets with highly similar stimulus characteristics across groups (PwMSD vs. PwMS: t = 1.26, p = 0.213, *β* = 0.19; PwMSD vs. HC: t = 0.56, p = 0.576; *β* = 0.08; PwMS vs. HC: t = -0.82, p = 0.408, *β* = -0.11). Please note that these statistics hold for valence as well as arousal due to the identical distribution of picture subsets across groups.

**MRI preprocessing**

Anatomical scans

*Manual lesion mapping/individual lesion masks*

A manual lesion mask was generated in a first step by experienced raters from the group of F.P. and under supervision of a neuroradiologist based on patients’ FLAIR images to determine the overall lesion volume, to facilitate Strategic LL determination, and for the combined spatial normalization and segmentation of T1-weighted MP-RAGE images described below.

*Combined spatial normalization and segmentation of T1-weighted MP-RAGE images*

In order to harness the high spatial resolution of anatomical T1-weighted MP-RAGE images for spatial normalization of fMRI images, we determined the spatial coregistration parameters mapping patients’ functional images from native space to the anatomical standard space defined by the MNI based on their anatomical T1-weighted MP-RAGE images. Specifically, in a first step, the FLAIR images and the corresponding lesion masks mentioned above were coregistered linearly to the native image space spanned by patients’ T1-weighted MP-RAGE images. In step two, probability voxel maps for GM, white matter (WM), and CSF were computed based on MP-RAGE images with the combined spatial normalization and segmentation algorithm. Voxel coordinates positioned in lesions as indicated by the coregistered lesion masks were ignored by the algorithm. This step produced tissue probability maps in the participant-specific/native image space and the MNI standard space. Standard-space images were adjusted for normalization-induced local deformations. Native image space probability maps were used to determine patients’ GM fractions (i.e., the number of GM voxels divided by the sum of all intracranial voxels). The deformation-corrected probability maps determined in MNI space (together with the lesion masks coregistered to MNI space) were used for determining GM, WM, and CSF group masks.

*Determination of group masks for GM, WM, and CSF*

Each voxel coordinate was assigned to one of the three tissues based on the maximal deformation-adjusted tissue probability for GM, WM, and CSF averaged across all 42 patients. Coordinates located in lesions as indicated by the coregistered lesion maps and the six direct neighbours of each lesion coordinate (in Euclidean distance to a lesion voxel of exactly one voxel) were classified as lesion tissue and did not enter any of the three group masks. The six neighbouring voxels of each lesion coordinate did also not enter any of the three group masks to account for potential partial voluming effects (cf. Weygandt et al., 2011).

*Visualization of amygdala and PFC coordinates evaluated*

Supplementary Figure 1 below illustrates the two regions of interest (ROI) evaluated in the present study.


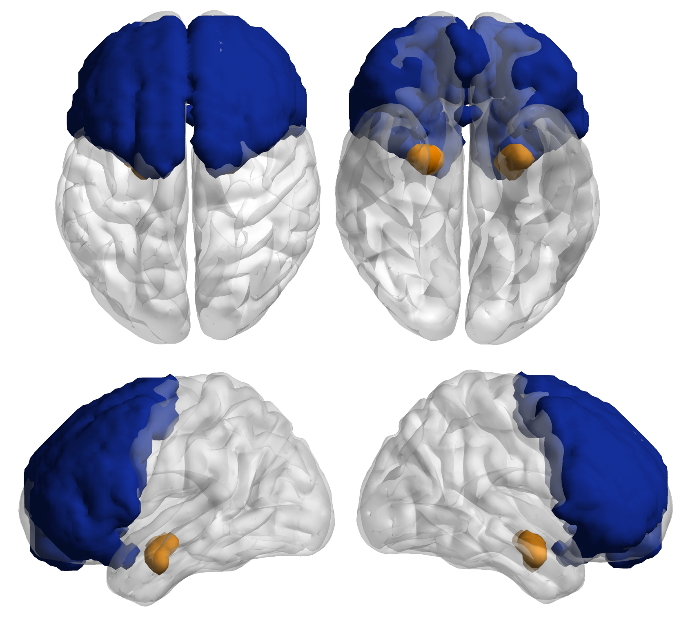


**Supplementary Figure 1** provides a volume rendering of amygdala and PFC coordinates evaluated in the study. The orange-coloured region corresponds to amygdala, the dark blue region to PFC. The individual regions underlying both larger regions were taken from the Neuromorphometrics brain atlas. Only coordinates simultaneously located in the atlas and the GM group mask (see above) were analyzed and thus visualized in this figure. The render brains in this and all other graphs in this work were generated with BrainNet (Xia et al., 2013).

Functional scans

Preprocessing of fMRI images comprised seven steps done using (toolboxes for) SPM12 (Wellcome Trust Centre for Neuroimaging, Institute of Neurology, UCL, London UK ­ <http://www.fil.ion.ucl.ac.uk/spm>). In first one, the 590 BOLD scans were corrected for patient head motion (yielding head motion parameters for each image). In step two, the two spin-echo EPI reference images with opposing phase encoding direction were coregistered to the average motion corrected BOLD image of a given patient. Next, the HySCO module from the SPM ACID toolbox (Ruthotto et al., 2012) and these two images were used to adjust the realigned fMRI images for potential inhomogeneities of the main magnetic field. Afterwards, BOLD images were corrected for slice acquisition order and linearly coregistered to the MP-RAGE images. In step six, the BOLD images were mapped to the anatomical standard space defined by the Montreal Neurological Institute (MNI; Tzourio-Mazoyer et al., 2002) using the spatial transformation parameters computed with the combined SPM12 segmentation and normalization algorithm based on patients MP-RAGE images (see *Combined spatial normalization and segmentation of T1-weighted MP-RAGE images*) with the SPM12 deformations toolbox. Finally, the BOLD images were smoothed in the spatial domain using an isotropic Gaussian kernel (full-width at half maximum of 8 mm). The resulting images were entered into fMRI group analyses*.*

Diffusion-weighted scans / tractography

Mapping of amygdala-PFC tracts was performed with Mrtrix3, necessary preprocessing steps with SPM12. In a first step, we used the ECMOCO module from the SPM ACID toolbox (Mohammadi et al., 2010) to correct the diffusion-weighted images (DWI) for head motion and eddy currents. In step two, two spin-echo EPI reference images with opposing phase encoding direction were coregistered to the DWI and the HySCO module from the SPM ACID toolbox (Ruthotto et al., 2012) and these two coregistered spin-echo EPI images were used to adjust the DWI for potential inhomogeneities of the main magnetic field. In the third step, the lesion mask of a given patient was coregistered to the DWI in order to identify lesion coordinates in the DWI. These lesion coordinates were discarded in the following fourth step, i.e., the non-linear spatial mapping of the anatomical standard space defined by the MNI to the native image space spanned by patients’ DWI images (i.e., inverse spatial normalization) using the SPM deformations toolbox. In the fifth step, the transformation parameters calculated in step four were used to map a couple of images to the native space of DWI images. In particular, the deformation-adjusted GM, WM, and CSF probability maps generated during processing of anatomical brain scans (see above), and masks for left amygdala, right amygdala, and PFC. These three masks were build using the Neuromorphometrics brain atlas (http://Neuromorphometrics.com; derived from the OASIS project http://www.oasis-brains.org), which is defined in MNI space and distributed with SPM12. To facilitate an anatomically constrained tractography of amygdala-PFC tracts (Smith et al., 2012) in the later step eight, we used the inversely normalized GM, WM, and CSF probability maps and the lesion mask defined in DWI space to generate a patient-specific mask of CSF in native DWI space in step seven. A voxel was considered CSF if it was more likely to belong to this tissue than to any other tissue and if it was not located in lesions as indicated by the lesion masks. Finally, in step eight, the tract connecting left amygdala and PFC as well as the one connecting right amygdala and PFC was computed separately with Mrtrix3 (Tournier et al., 2019). Specifically, coordinates located in the inversely normalized left / right amygdala were defined as potential seeds for this mapping and the mask of inversely normalized PFC as endpoint. As already suggested above, coordinates located in the CSF mask were defined as anatomical constraint, i.e., the algorithm was instructed to stop and discard a given streamline in case it crossed a CSF coordinate. In order to distribute amygdala-PFC streamlines in a comparable fashion across left and right amygdala, half of the 5000 streamlines identified by Mrtrix3 by default in each fibre tracking process were determined for left amygdala to PFC fibres, the other half for right amygdala to PFC fibres. After these streamlines were identified, we mapped them from streamlines to voxel space, which yielded the number of streamlines per voxel. Together with individual lesion masks defined in native DWI space, these number-of- streamlines -per-voxel maps were used to determine summary parameters of amygdala to PFC tract damage for each patient and evaluated in corresponding group analyses described below.

**Group analyses**

Psychological emotion regulation

*Control of MS modifying and antidepressant treatment*

To control a conceivable impact of MS modifying and antidepressant treatment on psychological emotion regulation (and emotional responsivity), we repeated the interaction analyses presented in the main text but evaluated only those patients, who (i) did received MS modifying treatment and (ii) did not receive an antidepressant treatment at the time of testing and within the criteria defined in the Participant section. This criterion was fulfilled by 28 patients. For 26 (9 PwMSD, 17 PwMS) of these 28 patients, rating data were available.

Given findings potentially suggesting that an interferon therapy applied to treat MS might pose a risk for developing depression (Pinto & Andrade, 2016), we additionally included a dichotomous CNI in the linear mixed models coding for the application of interferon treatment (y/n). As indicated by Tab. 1 in the main text, 9 MS patients received an interferon therapy.

Regional brain activity

*Brain activity related to emotional responsivity*

In order to underline the suitability of our fMRI task for measuring emotional responsivity, we performed proof-of-principle analysis testing main effects of this factor first. Specifically, one-sample t-tests were conducted based on voxel contrast difference maps “negative permit” minus “neutral permit”. Moreover, we also tested main effects of depression and effects of the interaction between depression and Strategic LL on brain activity reflecting emotional responsivity. Except for replacing voxel maps reflecting emotion regulation by those coding for emotional responsivity, all other parameters were as described for emotion regulation in the main text.

*Brain activity related to emotion regulation and emotional responsivity additionally controlled for putative treatment effects*

As for psychological emotion processing, we conducted the analysis testing for main effects of depression and of interaction effects of depression × Strategic LL on brain activity reflecting emotion regulation and emotional responsivity based on data of those 28 patients (9 PwMSD, 19 PwMS) who received MS modifying treatment but no antidepressant treatment. Again, we included a regressor coding for the application of interferons as additional CNI. We applied a more sensitive FWE-corrected significance threshold of a_FWE_ = 0.1 for the directed tests to account for the smaller sample size. Otherwise, all aspects were as described in the main text.

**Results**

**Group analyses**

Psychological emotion regulation

*Control of MS modifying and antidepressant treatment*

Results obtained for psychological emotion regulation (and emotional responsivity) obtained in an analysis controlling for treatment effects are summarized in Supplementary Figure 2 below.


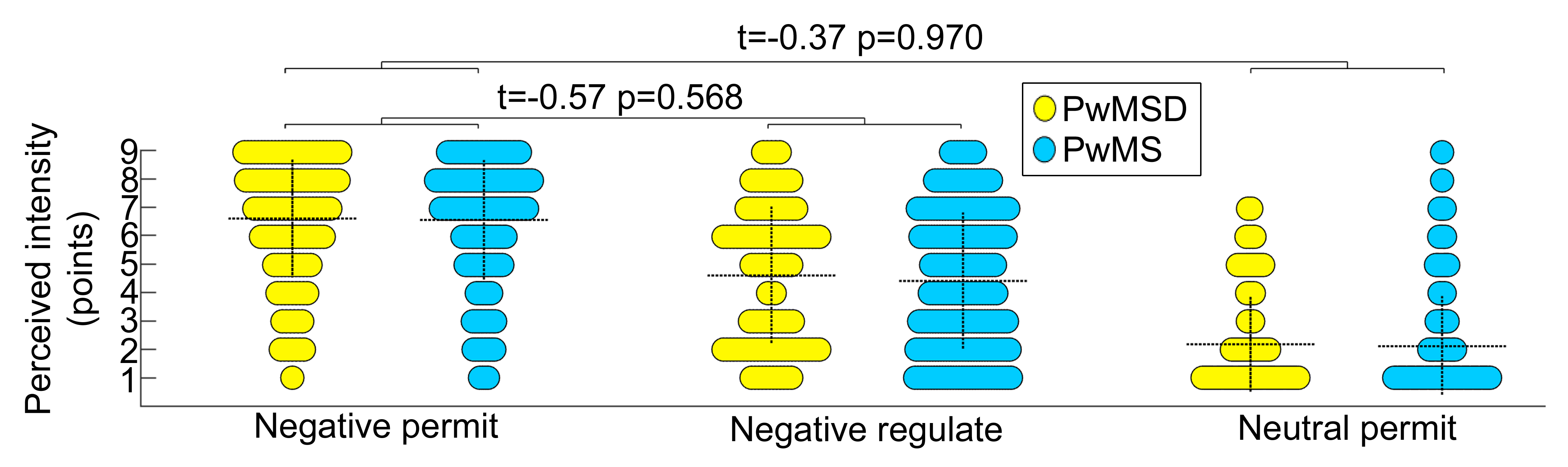


**Supplementary Figure 2.** The statistical parameters presented at each bracket correspond (from left to right) to the t-statistic, p-value. p-values correspond to false positive rates in directed t-tests. The dashed horizontal lines in each graph depict the mean, the dashed vertical lines the standard deviation for each combination of condition and group.­

Regional brain activity

*Brain activity related to emotional responsivity*

The below Supplementary Table 2 summarizes results obtained by repeating the functional MRI analyses described in the main text for modelling neural emotional responsivity instead of neural emotion regulation.

**Supplementary Table 2. Brain activity related to emotional responsivity.** x, y, z correspond to coordinates of voxels in the MNI-space with peak effect size in a cluster of significant voxels. The cluster size corresponds to the volume of significant voxels according to α_FWE_ = 0.05 in mm^3^. Abbreviations: CS - cluster size, i.e., the volume of significant voxels according to α_FWE_ = 0.05 in mm^3^; Param. - parameter; p_FWE_ – family-wise-error corrected false positive rate; x, y, z - coordinates of voxels in the MNI-space with peak effect size in a cluster of significant voxels. *Please note, that the regression coefficient for the constant intercept (i.e., the regressor of interest in this analysis) cannot be standardized due to its lack in variation. Consequently, we cannot report the standardized regression coefficient as effect size measure in this proof-of-principle analysis.

| **Effect / Region** | **x** | **y** | **z** | **CS** | **t** | **p_FWE_** | **β** |
| --- | --- | --- | --- | --- | --- | --- | --- |
| Emotional responsivity |  |  |  |  |  |  |  |
| Amygdala | 27 | -3 | -18 | 378 | 4.98 | 2 · 10^-4^ | * |
| Amygdala | -21 | -3 | -15 | 324 | 4.30 | 0.002 | * |
| Superior frontal gyrus | -6 | 63 | 21 | 864 | 5.17 | 0.003 | * |
| Inferior frontal gyrus | 51 | 42 | 6 | 297 | 5.13 | 0.003 | * |
| Inferior frontal gyrus | -45 | 39 | 12 | 54 | 4.82 | 0.008 | * |
| Superior frontal gyrus | 6 | 54 | 24 | 27 | 4.28 | 0.032 | * |
| Superior frontal gyrus | 6 | 63 | 21 | 54 | 4.23 | 0.036 | * |
| Superior frontal gyrus | -9 | 54 | 39 | 27 | 4.11 | 0.048 | * |
|  |  |  |  |  |  |  |  |
| Depression ×  Strategic LL |  |  |  |  |  |  |  |
| Superior frontal gyrus | 15 | 3 | 72 | 351 | 5.31 | 0.005 | 0.65 |
| Inferior frontal gyrus | 36 | 30 | 6 | 189 | 5.15 | 0.007 | 0.67 |
| Inferior frontal gyrus | 57 | 15 | 6 | 108 | 5.01 | 0.010 | 0.66 |
| Superior frontal gyrus | -6 | 63 | 6 | 162 | 4.79 | 0.017 | 0.64 |
| Superior frontal gyrus | -9 | 33 | 27 | 27 | 4.62 | 0.024 | 0.62 |

*Brain activity related to emotion regulation and emotional responsivity additionally controlled for putative treatment effects*

As in the analysis presented in the main text not controlling for treatment effects, an interdependent impact of the depression and Strategic LL on emotion regulation was found in left amygdala. Please note, that this voxel cluster (peak coordinate MNI: -18, -6, -15) also included coordinate MNI: -18, -9, -15 (i.e., the peak coordinate found in the corresponding analysis in the main text). See Supplementary Table 3

**Supplementary Table 3.** Abbreviations: *β -* standardized regression coefficients or effect size respectively; CS - cluster size, i.e., the volume of significant voxels according to α_FWE_ = 0.1 in mm^3^. Param. - parameter; p_FWE_ – family-wise-error corrected false positive rate; x, y, z - coordinates of voxels in the MNI-space with peak effect size in a cluster of significant voxels.

| **Param. / Effect / Region** | **x** | **y** | **z** | **CS** | **t** | **p_FWE_** | **β** |
| --- | --- | --- | --- | --- | --- | --- | --- |
| Emotion regulation |  |  |  |  |  |  |  |
| Depression |  |  |  |  |  |  |  |
| Superior frontal gyrus | -24 | 51 | 6 | 27 | -4.35 | 0.089 | -0.70 |
|  |  |  |  |  |  |  |  |
| Depression × Strategic LL |  |  |  |  |  |  |  |
| Amygdala | -18 | -6 | -15 | 54 | 3.04 | 0.049 | 0.50 |

**References**

Mohammadi S, Moller HE, Kugel H, Muller DK, Deppe M. Correcting eddy current and motion effects by affine whole brain registrations: evaluation of three-dimensional distortions and comparison with slicewise correction. Magn Reson Med 2010; 64: 1047 – 56.

Pinto EF, Andrade C. Interferon-related depression: A primer on mechanisms, treatment, and prevention of a common clinical problem. Curr. Neuropharm. 2016; 14: 743 – 8.

Ruthotto L, Kugel H, Olesch J, Fischer B, Modersitzki J, Burger M, et al. Diffeomorphic Susceptibility Artefact Correction of Diffusion-Weighted Magnetic Resonance Images. Physics in Medicine and Biology. 2012; 57: 5715-31.

Smith RE, Touriner JD, Calamante F, Connelly A. Anatomically constrained tractography: improved diffusion MRI streamlines tractography through effective use of anatomical information. NeuroImage. 2012; 62: 1924 – 38.

Tournier D, Smith R, Raffelt D, Tabbara R, Dhollander T. MRtrix3: A fast, flexible and open software framework for medical image processing and visualization. NeuroImage. 2019; 202: 116 – 137.

Tzourio-Mazoyer N, Landeau B, Papathanassiou D, Crivello F, Etard O, Delcroix N. Automated anatomical labeling of activations in SPM using a macroscopic anatomical parcellation of the MNI MRI single-subject brain. Neuroimage. 2002; 15: 273 – 89.

Weygandt M, Hackmack K, Pfüller CF, Bellmann-Strobl J, Paul F, Zipp F, Haynes JD. MRI Pattern Recognition in Multiple Sclerosis Normal-Appearing Brain Areas. PLoS ONE 2011. 6: e21138.

Xia M, Wang J, He Y (2013) BrainNet Viewer: A Network Visualization Tool for Human Brain Connectomics. PLoS ONE 8: e68910
